# Comparison of peripherally inserted central catheters (PICCs) versus totally implantable venous-access ports in pediatric oncology patients, a single center study

**DOI:** 10.1038/s41598-022-07584-8

**Published:** 2022-03-03

**Authors:** Hong Zhang, Yumei Li, Nannan Zhu, Yanfang Li, Jinqiu Fu, Jing Liu

**Affiliations:** 1 Department of Pediatrics, Qilu Hospital, Shandong University, No.107, West of Wenhua Road, 250012 Jinan, People’s Republic of China; 2 School of Public Health, Cheeloo College of Medicine, Shandong University, Jinan, 250012 People’s Republic of China

**Keywords:** Haematological cancer, Paediatric cancer

## Abstract

To compare the efficacy of peripherally inserted central catheters (PICCs) and totally implantable venous-access ports (TIVAPs) for chemotherapy of pediatric patients with malignant tumors. A total of 96 children with malignant tumors who received catheterization of PICCs or TIVAPs for chemotherapy from May 2020 to May 2021 in Department of Pediatric Oncology of Qilu Hospital of Shandong University were selected. Then, the pathological features of disease, the age of children, the indwelling time, the incidence of postoperative complications, and the satisfaction degree were compared between the two groups. The age of children in the TIVAP group was younger than that in the PICC group (*P* < 0.05). The indwelling time in the TIVAP group was 7.2 ± 2.757 months,which was significantly longer than 5.65 ± 2.058 months in the PICC group (*P* < 0.05). The incidence of postoperative complications in the TIVAP group without systemic or local infection was markedly lower than that in the PICC group (*P* < 0.05). The satisfaction degree of patients in the TIVAP group without unsatisfied was markedly higher than that in the PICC group (*P* < 0.05). TIVAPs may be the first choice for chemotherapy of children with malignant tumors.

## Introduction

The incidence of childhood cancer has increased over the past few decades worldwide^1–6^. Cancer is the second most common cause of death in children who were aged 0–14 years old. The most common types of childhood cancers include leukemia, brain cancer, lymphoma, and other solid tumors^6^. Although the overall survival rate of children with acute lymphoblastic leukemia (ALL) is more than 90%, leukemia remains one of the leading causes of death^7^. Fortunately, increasing knowledge on biology and tumorigenesis of childhood cancer contributes to advance in chemotherapy, supportive care, and personalized medicine^7^. However, cancers, especially childhood cancers, are currently treated by chemotherapy, and a long-term venous access is essential for the children with malignant diseases.

Central venous catheters (CVCs) reduce the stimulation to the vein and skin and improve injection of liquid medicines, which are usually used for patients receiving chemotherapeutic drugs^8^. Both peripherally inserted central catheters (PICCs) and totally implantable venous-access ports (TIVAPs) are frequently used for chemotherapy of children with cancer^9^. It has been shown that PICCs possess various advantages, such as convenient placement without pleura-pulmonary damage, low cost, and long-term and stable vein access^10^. At present, PICCs are extensively utilized in clinical management of chemotherapy, medication administration, and parenteral nutrition. For pediatric patients, due to vascular conditions, their own distress, non-cooperation and other reasons, the application of PICCs is limited, however, TIVAPs are a compensation for its deficiency. TIVAPs assist patients with a safe and permanent access to a vein, which are often used in patients who need continuous administration of intravenous drugs, including those receiving chemotherapy^12^. TIVAPs are also used for the purposes of regular intravenous medications, transfusion of blood products, parenteral nutrition, or the necessity of regular periodic blood sampling^13^. This retrospective study aimed to compare the efficacy of PICCs and TIVAPs for chemotherapy of pediatric patients with malignant tumors.

## Results

### Childhood retrospective cohort: a case–control study of childhood cancers treated with PICCs or TIVAPs

**Figure 1:**
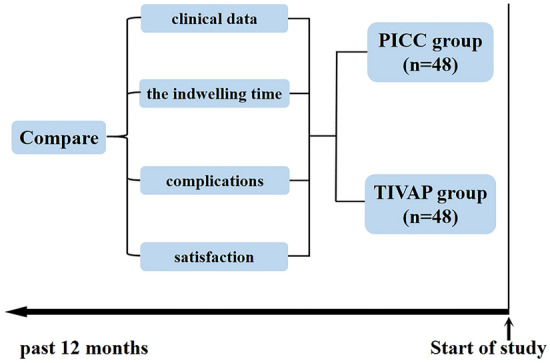
An overview of the study.

An overview of the study is shown in Fig. 1. 96 childhood patients with cancers who were treated with PICCs or TIVAPs from May 2020 to May 2021 were retrospectively analyzed. A timeline of the study is shown in Fig. 1.

### Patients’ demographic and clinical data

**Figure 2:**
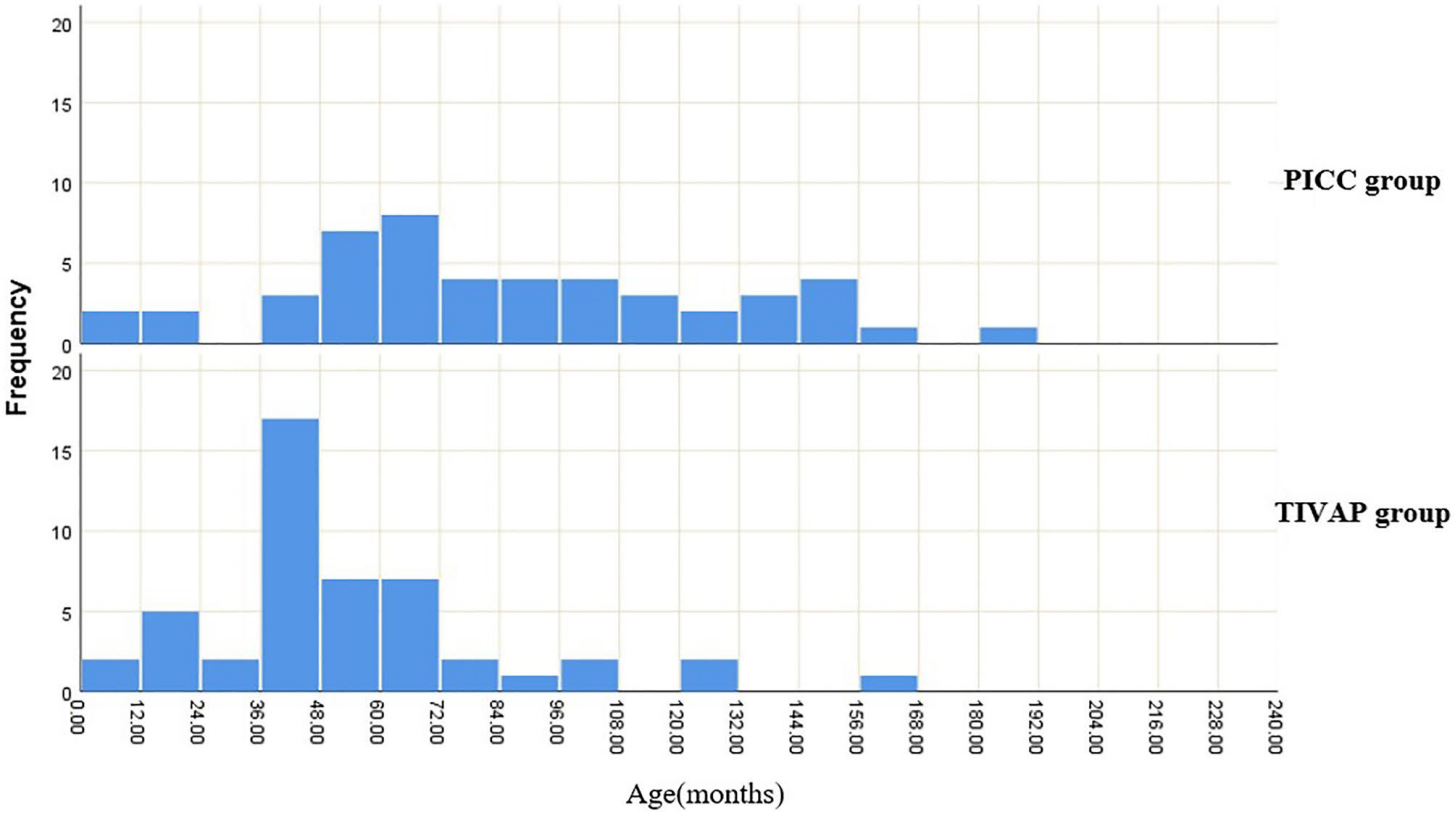
The frequency of age in PICC group and TIVAP group.

Patients’ age in the TIVAP group was significantly younger than that in the PICC group (*P* < 0.05). The frequency of age in two groups is shown in Fig. 2. There was no significant difference between the two groups in terms of gender and pathological features of the disease (*P* > 0.05) (Table 1).

**Table 1:** General information [n (%)] (mean ± SD).

	TIVAP group(n = 48)	PICC group(n = 48)	*P* value
Age(months)	47 ± 4.358	78 ± 6.066	0.000
**Sex**			0.519
Male	30	33	
Famale	18	15	
**Clinical pathological types**			0.093
Leukemia	38	28	
Lymphoma	6	7	
Immature teratoma	1	0	
Neuroblastoma	1	2	
Rhabdomyosarcoma	1	2	
Hepatoblastoma	1	2	
Nephroblastoma	0	3	
Intracranial tumours	0	4	

### The indwelling time

**Figure 3:**
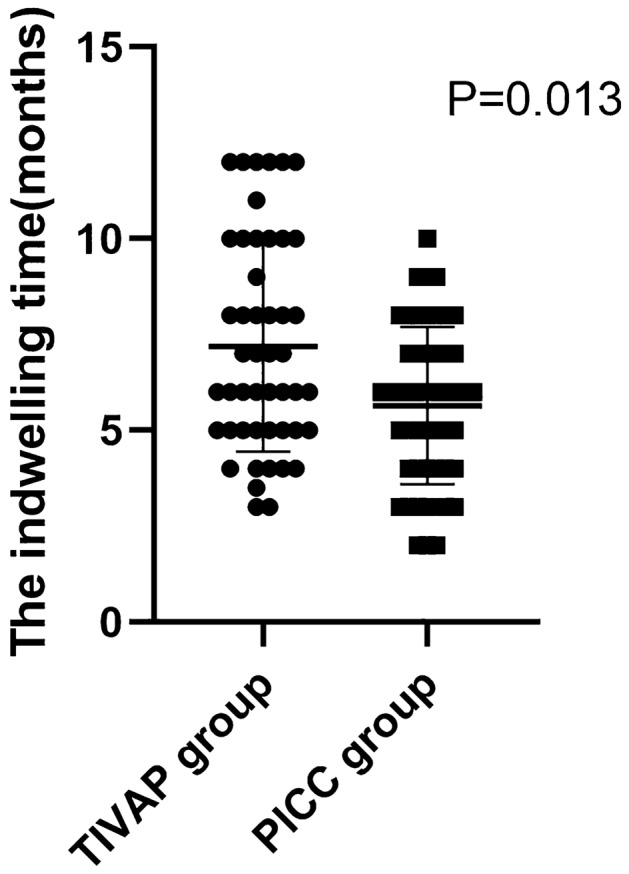
The comparison of the indwelling time in the TIVAP group and in the PICC group. The indwelling time was calculated monthly. The indwelling time in the TIVAP group is significantly longer than that in the PICC group (*P* < 0.05).

The indwelling time was 7.2 ± 2.757 months in the TIVAP group, which was significantly longer than 5.65 ± 2.058 months in the PICC group (*P* < 0.05) (Fig. 3).

### Postoperative complications

In the PICC group, there were 21 cases with local infection, 11 with phlebosclerosis, 11 with local allergy, 3 with local bleeding, 1 with thrombogenesis, 1 with catheter displacement, and 9 with catheter obstruction. In the TIVAP group, there were only 9 cases with catheter obstruction. The incidence of postoperative complications including local infection, phlebosclerosis, and local allergy in the TIVAP group was markedly lower than that in the PICC group (*P* < 0.05) (Table 2). Moreover, with any complications as dependent variables and gender, age, pathological features, indwelling time as independent variables, logistic regression was performed. It was found that compared with PICC group, TIVAP group had a lower incidence of complications, with OR = 0.000 (95%CI 0.000–0.132), and long indwelling time may be a risk factor of complications (*P* = 0.021) after adjusting the factors of gender, age, pathological type and indwelling time (Table 3).

**Table 2:** Comparison of complications [n (%)].

Groups complications	Local infection	Phlebophlogosis	Local allergy	Local blooding	Thrombogenesis	Catheter displacement	Catheter obstruction	Any
TIVAP group	0	0	0	0	0	0	9(18.75)	9(18.75)
PICC group	21(43.75)	11(22.92)	11(22.92)	3(6.25)	1(2.08)	1(2.08)	9(18.75)	43(89.58)
χ2 value	26.880	12.424	12.424	1.376	–	–	–	48.503
*P*-value	0.000	0.000	0.000	0.241	1.000(Fisher's exact test)	1.000(Fisher's exact test)	1.000(Fisher's exact test)	0.000

**Table 3:** Multivariate regression analysis for complications and satisfaction.

Variable	Complications			Satisfaction		
	95% CI	Exp(B)	*P*-value	95% CI	Exp(B)	*P*-value
Disposal methods (PICC vs. TIVAP)	0.000–0.132	0.000	0.008	0.018–0.791	0.119	0.028
Gender (male vs. famale)	0.383–5.199	1.411	0.605	0.016–1.160	0.136	0.068
Age (months)	0.983–1.021	1.002	0.871	0.978–1.016	0.997	0.749
Indwelling time (months)	1.046–1.732	1.346	0.021	0.817–1.402	1.070	0.622
Clinical pathological types			0.512			0.648
Leukemia vs. lymphoma	0.063–2.858	0.425	0.379	0.035–3.341	0.342	0.357
Leukemia vs. others	0.222–15.675	1.864	0.567	0.141–4.224	0.773	0.766

### Catheter-related satisfaction

Satisfaction questionnaire was implemented in the two groups. In the PICC group, 38 cases were highly satisfied, 9 were moderately satisfied, and 1 was not satisfied. In the TIVAP group, 46 cases were satisfied, of whom 1 case was moderately satisfied. No patient was found unsatisfied in the TIVAP group. All questionnaires without complications were "satisfied", so the impact of complications on satisfaction was not considered in the multivariate regression analysis. With satisfaction (Satisfied = 1, basically satisfied or not satisfied = 2) as dependent variables and gender, age, pathological features, indwelling time as independent variables, logistic regression was performed, and it was found that the satisfaction degree of children in the TIVAP group was significantly higher than that in the PICC group OR = 0.119 (95%CI 0.018–0.791) after adjusting the factors of gender, age, pathological type, indwelling time and complications (Table 3).

## Discussion

As childhood malignant tumors that have an increasing incidence rate, a high recurrence rate or high persistence but a low mortality rate, leukemia or other solid cancers have different histological types and subtypes, as well as cell sources, characteristics and prognoses. Chemotherapy and laboratory tests of blood are necessary for these childhood patients. CVCs allow safe administration of intravenous cytotoxic medications, facilitate the administration of intravenous fluid resuscitation or parenteral nutrition, and help blood collection for relieving the pain. CVCs have been largely used in oncology department in China^14^. In addition, CVCs can protect peripheral veins and support chemotherapy with significant advantages compared with peripheral vein catheters^15^. In the present study, all CVCs were used for chemotherapy. This study compared the efficacy of TIVAPs and PICCs for chemotherapy of children with cancer.

TIVAPs not only assist children with a safe vascular access for treatment, but also protect vessels from repeated puncture injuries^16^. According to our experience, in children, the younger the patients are, the greater the advantages of TIVAPs will be. The distance from PICC to the venous tube is slightly longer, which may increase the risk of traction and infection due to children's activities. The catheter is observable out of the body, children’s clothing is partially limited, the exposed catheter is easy to prolapse or to be accidentally pulled out which is associated with catheter displacement, and it may also be accompanied by leakage during activities or hemorrhage. However, an implantable port is fully implanted subcutaneously without any exposed part and cannot be removed by itself. Besides, the distance to the tube is shorter than that of PICC, thus, the risk of infection is reduced^17^. Patel et al.^9^ and Taxbro et al.^11^ reported a lower incidence of postoperative complications for TIVAPs compared with PICCs in randomized controlled trials. Therefore, it is appropriate for children with hyperactivity and a strong sense of curiosity. Although not statistically significant, there seems a difference in the cancer types between TIVAP and PICC, which may be explained by the treatment cycles of the cancer types. The longer the treatment period, the more likely TIVAP because of its long lifespan.

In the current study, the longest indwelling time was 7.2 ± 2.757 months in the TIVAP group, while that was 5.65 ± 2.058 months in the PICC group. According to the guidelines, the indwelling time in PICC group was 12 months, while that was 240 months in the TIVAP group (in theory). However, in our ward, the indwelling time in the PICC group was about 8 months, and no TIVAP was taken out from patients. For the treatment of children with ALL, the total course of disease is about 30 months, while that is within 12 months in the majority of cases with solid tumors. Thus, patients requiring long-term treatment are better candidates for TIVAPs, which is consistent with the previous findings^16^.

TIVPA was found to be associated with postoperative complications, and it was related to a higher satisfaction degree of patients compared to PICC in the present study, which is in agreement with the result of another research^18,19^. While CVCs are clinically popular, their complications, especially the serious complication of infections are still common and considered to cause damage to patients, which are associated with morbidity, mortality, and financial costs of the patients. At present, the prevention of CVCs-related injuries served by multidisciplinary team involvement is taken into consideration for healthcare researchers, clinicians and patients. Local infection is manifested as redness, swelling, heat and pain at the puncture site, while local allergy is manifested as red rashes with itchy sensation. In the present study, there were more local infections and local allergies in the PICCs group, which may be related to the local application of the plastic sticking dressings. Phlebophlogosis with or without thrombogenesis, a relatively common peripheral vascular disease, is aseptic inflammation of the veins. Standard practice requires PICC catheter insertion in the basilic vein, which is 0.4–0.5 cm in diameter compared to TIVAP catheter insertion into the subclavian vein, which is 1–2 cm in diameter. Although the lumens of both catheters are similar, the smaller diameter of the basilic veins used for the PICC catheter is likely to result in slower blood flow rates, increasing the risk of blood clot formation. On the other hand, the longer intravenous length of a PICC catheter increases the surface area for the propagation of thrombosis and catheter obstruction, while the TIVAP catheter tip without an anti-reflux device may explain the occurrence of catheter obstruction.

The major limitation of this study was the lack of cost analysis in the two groups. In some practice settings, TIVAP are currently the standard CVC in oncology. In our practice location, a 50–50 split between TIVAP and PICC may be related to cost or the risk of surgical procedures. There were direct and indirect costs, and direct costs included medical and non-medical costs. Medical costs covered costs related to drugs, surgery, laboratory tests, non-laboratory tests, and medical consumptive costs (e.g., a hospital’s expenses). Non-medical expenses included costs related to transportation, accommodation, and nutrition of children and their staffs. An indirect economic burden included the economic loss caused by the loss of working opportunities^20^. A study showed that there was no significant difference in medical expenses if a CVC would be still in place at 6 months after insertion^9^. Another study indicated that when the period of treatment was longer than 12 months, there was no significant difference in medical costs between TIVAP and PICC groups^21^. Thus, the daily expenses for CVCs were highly associated with the indwelling time, which was significantly longer in the TIVAP group. Another limitation of this study was non-randomized nature and inadequate sample size, which was mainly due to the limited number of patients at a single center. An inadequate sample size may have led to the lack of statistical significance in the time to the first major complication observed. Prospective randomized controlled studies with large sample size are required to further verify our findings.

## Conclusions

In summary, TIVAPs were associated with a longer indwelling time and a lower incidence of postoperative complications, thereby confirming their higher efficiency compared with PICCs. TIVAPs are recommended for children with malignant tumors, in particular those requiring long-term treatment, and health-based educational programs are also recommended during implantation, so as to reduce the risk of postoperative complications and to improve the satisfaction rate of families of children with cancer. However, further prospective studies are required to validate our findings and to ascertain medical expenses and the incidence of postoperative complications, in order to better highlight the cost-effectiveness of CVCs.

## Patients and methods

### Subjects

Clinical data of 96 patients with cancer who were treated with PICCs or TIVAPs in Department of Pediatric Oncology of Qilu Hospital of Shandong University (Jinan, China) from May 2020 to May 2021 were retrospectively analyzed.

#### Statement

PICCs method was carried out in accordance with nursing practice standards for intravenous therapy. TIVAPs method was carried out in accordance with Expert consensus and Technical Operation Guide for Clinical Application of TIVAPs (2017 edition). Authors confirm that all experimental protocols were approved by Ethics Committee of Shandong University Qilu Hospital. The research involving human research participants have been performed in accordance with the Declaration of Helsinki.

#### Inclusion criteria

Patients who were diagnosed by bone marrow and peripheral hemocytological changes or clinicopathology; patients who were aged < 18 years old and had no history of mental disorders; patients who were conscious; patients with complete clinical data; signed written informed consent form by patients’ parents before the study.

#### Exclusion criteria

Patients with multiple organ failure; patients with a poor compliance.

### Methods

The Seldinger technique was used in both groups ^22^. A detailed description of the methods of TIVAPs and PICCs can be found in the literature^9,21^.

### Observational indices

The pathological features of disease, the age of children, the indwelling time, the incidence of postoperative complications, and the satisfaction degree were compared between the two groups. The pathological features of disease and the age of children were identified in the two groups. The indwelling time was calculated monthly. Until the time of the study, if the catheter was removed, the indwelling time was calculated; if the catheter was not removed, the indwelling time was calculated as the time from the catheter insertion date to the study date. The satisfaction degree was assessed through a questionnaire, which was sent to patients' parents three months after catheterization with anonymous responses covering purpose, maintenance, management of postoperative complications, and patients’ attitudes toward catheters and staffs’ perceptions. The survey question 9,10 and 12 provided the data reported. The remaining questions were used to evaluate the management of maintenance. The designed questionnaire is presented in Supplementary information file.

### Statistical analysis

The SPSS 21.0 (IBM Corp., Armonk, NY, USA) and GraphPad Prism 8.0 (GraphPad Software Inc., San Diego, CA, USA) software were used to perform statistical analysis and graphical illustration, respectively. Categorical variables were expressed as percentages, and Chi-square test was used to compare data between two groups. Measured data were expressed as mean ± standard deviation (SD). *P* < 0.05 was considered statistically significant.

## Supplementary Information

Supplementary Information.
