# Supplementary material for: Comparison of peripherally inserted central catheters (PICCs) versus totally implantable venous-access ports in pediatric oncology patients, a single center study
Source: Sci Rep. 2022 Mar 3;12:3510. doi: 10.1038/s41598-022-07584-8 (PMC8894364; doi:10.1038/s41598-022-07584-8)
Supplement: Supplementary file 1 — Supplementary Information. [file 41598_2022_7584_MOESM1_ESM.pdf]

Satisfaction survey of children with PICC

Dear family members,

Hello!Thank you for your trust in our hospital. In order to understand the medical service situation of our department, make our work improve constantly and more close to your needs, please kindly inform us of the service situation of the medical staff truthfully (please tick √ before the agreed items).Thank you for your cooperation. I wish you recover soon.

Section: number: time:

1. Are you clear about the purpose of PICC?
Clear () basically clear () not clear ()
2. Are you clear about the matters needing attention during the period?
Clear () basically clear () not clear ()
3. Does your nurse explain the matters needing attention during this period ?
Yes () No ()
4. Are you clear about the maintenance instructions of PICC?
Clear () basically clear () not clear ()
5. Do you know the common complications during this period?
Yes () No()
6. Are you satisfied with the nurse's service attitude?
Satisfied () Basically satisfied () Not satisfied ()
7. Are you satisfied with the nurse's maintenance techniques?
Satisfied () Basically satisfied () Not satisfied ()
8. When you encounter problems, can the nurse help you solve them patiently?
Can () basic can () cannot ()
9. Are you satisfied with the time of PICC?
Satisfied () Basically satisfied () Not satisfied ()
10. Are you satisfied with the operation technology of PICC?
Satisfied () Basically satisfied () Not satisfied ()
11. Do you think the inspection after PICC is reasonable?
Reasonable () unreasonable ()
12. Are you satisfied with the application in our department?
Satisfied () Basically satisfied () Not satisfied ()
